# Applicability of Single-Camera Photogrammetry to Determine Body Dimensions of Pinnipeds: Galapagos Sea Lions as an Example

**DOI:** 10.1371/journal.pone.0101197

**Published:** 2014-07-02

**Authors:** Kristine Meise, Birte Mueller, Beate Zein, Fritz Trillmich

**Affiliations:** Department of Animal Behaviour, University of Bielefeld, Bielefeld, Germany; University of Tasmania, Australia

## Abstract

Morphological features correlate with many life history traits and are therefore of high interest to behavioral and evolutionary biologists. Photogrammetry provides a useful tool to collect morphological data from species for which measurements are otherwise difficult to obtain. This method reduces disturbance and avoids capture stress. Using the Galapagos sea lion (*Zalophus wollebaeki*) as a model system, we tested the applicability of single-camera photogrammetry in combination with laser distance measurement to estimate morphological traits which may vary with an animal’s body position. We assessed whether linear morphological traits estimated by photogrammetry can be used to estimate body length and mass. We show that accurate estimates of body length (males: ±2.0%, females: ±2.6%) and reliable estimates of body mass are possible (males: ±6.8%, females: 14.5%). Furthermore, we developed correction factors that allow the use of animal photos that diverge somewhat from a flat-out position. The product of estimated body length and girth produced sufficiently reliable estimates of mass to categorize individuals into 10 kg-classes of body mass. Data of individuals repeatedly photographed within one season suggested relatively low measurement errors (body length: 2.9%, body mass: 8.1%). In order to develop accurate sex- and age-specific correction factors, a sufficient number of individuals from both sexes and from all desired age classes have to be captured for baseline measurements. Given proper validation, this method provides an excellent opportunity to collect morphological data for large numbers of individuals with minimal disturbance.

## Introduction

Morphological features such as body size and mass have been shown to correlate positively with many life history traits such as lifespan and fertility ([1]–[4]). In polygynous species, male reproductive success is expected to increase with male body size and mass ([5],[6]) which have been selected for increased fasting ability ([2],[7]) or advantages in intra-sexual competition for access to females ([8],[9]). Furthermore, body length and mass have been used as indicators to assess habitat quality (e.g., [10]) as an individual’s body condition provides information about the quantity and quality of resources in a habitat (e.g., [11], [12]). Therefore, determining different body dimensions is of high interest for biologists studying behavioral, evolutionary, or ecological questions.

In the wild, direct physical measurements of body length or mass are often difficult to obtain. Measurements from physically restrained animals are limited by the size and strength of the animal. Anaesthesia enables accurate measurement, but can be risky for the animals as it may cause stress, injuries, or even death (e.g., in marine mammals, [13]). Furthermore, anaesthetizing animals is impractical if measurements must be taken for large numbers of animals or repeatedly over time. Accordingly, a growing number of studies have assessed the possibility to obtain size and mass estimations indirectly by photogrammetry (e.g., *Mirounga leonina*: [14], *Gorilla gorilla*: [15], *Physeter macrocephalus*: [16], *Loxodonta Africana*: [17], *Macaca fuscata*: [18], *Capra ibex*: [19], *Orcinus orca*: [20]).

Photogrammetry has been shown to provide useful, highly accurate estimates of an individual’s body length and body mass (e.g., [14], [21],[22]). Two different approaches can be distinguished: stereo- and single-camera photogrammetry. The first is based on multiple photographs which are used to create 3D-models of the animals (e.g., [23], [24]). In single-camera photogrammetry only one photograph is needed. The animal is scaled by either providing an object of known length ([25], [26]), using two parallel lasers with a fixed distance ([22]), or estimating the distance between the object and the camera, (e.g., with a laser distance meter [16]).

Photogrammetric methods differ in their efficiency when taking into account the time needed to collect and analyze the data and the potential disturbance to the animals. If a scale needs to be in the photograph, two measurements per photograph must be taken: one for the scaling object and a second one of the individual’s body length. Moreover, to position measuring poles of known length or obtain 3D-models using stereo-photogrammetry, animals have to be approached closely (from different directions) which often proves to be difficult and likely causes disturbance. In single-camera photogrammetry often two photographs are taken to assess body mass: one laterally, and the other either from the front or the back end of the animal (e.g., [21], [25]). Again, this might prove to be difficult to achieve when studying highly mobile or shy species.

Here we present a modification of the single camera photogrammetric method developed by Jaquet ([16]). This method has been used to estimate body mass of sperm whales via the length of their flukes. An object of a known length was photographed from different distances and the number of pixels corresponding to the length of the object was measured. By regressing the number of pixels against the distance, Jaquet ([16]) derived an equation which could be used to calculate the fluke span from photographs taken from a known distance. This technique provides astonishingly accurate results even at large distances. Given that a whales’ fluke is fairly rigid, we wanted to expand the single-camera photogrammetric method to a situation where the trait of interest presents itself in a more variable manner. This is the case for sea lions, which often rest in a wide range of different body positions. Furthermore, we wanted to assess if it was possible to estimate body mass based on morphological traits which can readily be estimated by photogrammetry. In right whales, photogrammetry has also been used to assess changes in body shape of lactating and non-lactating females ([27]). Thus, we applied single-camera photogrammetry to estimate body length, axillary girth and body mass of adult Galapagos sea lions. Although this is the smallest sea lion species in the world, adult females have a standard body length of 156 cm (max. 176 cm) and weigh at maximum 100 kg ([28]). It is not always feasible to obtain body measurements by capturing animals. For example, females coming ashore might be pregnant or nursing and capturing would increase the risk of abortion or abandonment of pups. Male Galapagos sea lions can reach a body length of 210 cm and may weigh up to 200 kg during the reproductive season ([28]). Due to their high body mass, only non-territorial males which have not yet reached their final body size have been captured. Even here, anaesthesia was needed to physically measure the body mass of the largest captured males (max. 158 kg). The photogrammetric method described here allows researchers to obtain accurate estimates of body length and useful estimates of body mass of resting individuals without disturbance of the animals or risk to the investigator.

## Methods

The study was conducted on Caamaño, a small islet in the centre of the Galapagos archipelago (0°45′ S. 90°16′ W), which harbours a large breeding colony of Galapagos sea lions. Many individuals could be identified by numbered tags (Allflex sheep ear tags of size 0, UK) applied to the trailing edge of both front flippers; others were marked by preliminary bleaches of their fur. Data were collected between September and December 2003–2012 for measurements and 2008–2012 for photogrammetric estimates. Captures were performed using hoop nets (Fuhrman Diversified, USA; R. Lohmann tailoring, Germany) and animals were manually restrained. Morphometric measurements taken during most captures included standard nose-to-tail straight body length, axillary girth (to the nearest 0.5 cm) and body mass (Kern HUS 300K100, ±0.1 kg, Table 1). Photographs were taken using a digital camera (Canon EOS D300) fitted with a Canon EFS 18–55 mm zoom lens. Focal length was fixed at 55 mm. At this focal length it has negligible pincushion distortion (0.42%). All photographs were taken at approximately 90° angle to the longitudinal axis of the animal. Simultaneously, a handheld laser distance meter (Leica Disto™ A5) firmly attached to the camera was used to measure the distance between camera and the focal animal. During the study period, a total of 87 adult individuals (37 males, 50 females) were captured and photographed within the same season.

**Table 1:** Minimum and maximum values of the morphometric measurements taken during captures of individuals for which photogrammetric data existed.

morphometric data	males (N = 37)	females (N = 50)
body length	118–192 cm	122–169 cm
girth	82–107 cm	75–101 cm
body mass	52–98 kg	43–88 kg

### Calibration

To relate the distance between object and camera to the body length of the animal we first calibrated the camera and lens ([16]). We photographed an object of one meter length at ranges from 2.5 to 15 m (N = 65 photographs). We measured the number of pixels that matched the length of the object using the measuring tool of the program Image J 1.42, a Java-based image processing software. These data were used to calculate a linear regression function (object length/number of pixels = a*distance – b). The derived equation.
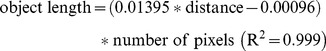
(1)was used to calculate the body length of the focal animal.

**Figure 1:**
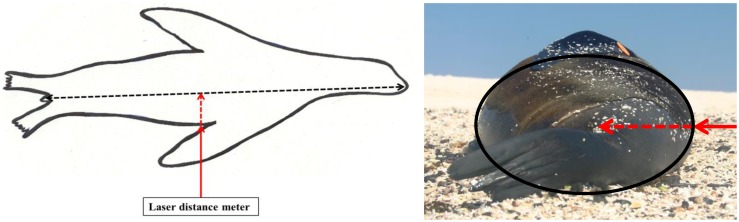
Systematic error in distance measurements. The laser distance meter measured the distance between camera and the first contact with the animal in the periphery of the body (solid red line). Because length measurements were taken at the body centre (dashed black line), the measured distance does not equal the distance between camera and measurement point (red solid and dashed line).

A systematic error emerged through errors in distance measurements. Due to the animals’ cylindrical body shape, measured distances are slightly shorter than the distance to the midline of the animals where the body length measurements were taken (Figure 1). Ignoring this fact leads to systematic underestimation of the animals’ standard body length. To deal with this problem we added the missing distance, estimated as girth/2π, to the measured distance for the subsequent calculation of individual body length. As girth measurements are only available for captured individuals, we compared capture data with photogrammetric data and developed a correction factor that can be used to obtain real body length from photogrammetric data (see below).

### Data analysis

**Figure 2:**
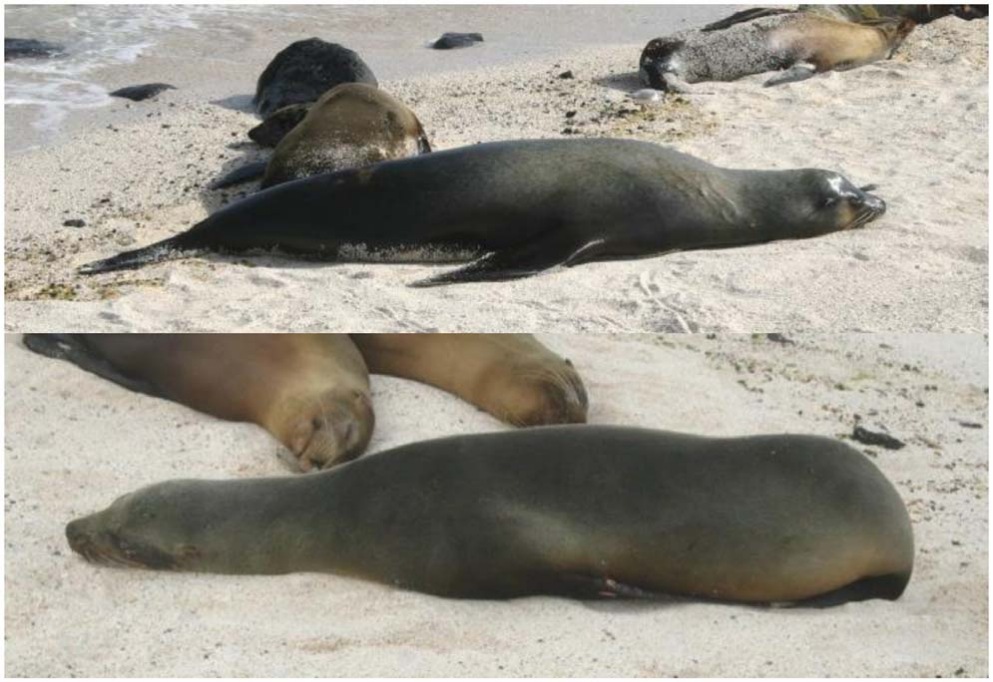
Categorization of photographs according to the animals’ body position. Photographs were assigned to different categories depending on the position of the animal in the photograph. The upper photograph shows a well-stretched out animal (photograph category 1) while the animal on the lower photograph has its hind flippers tucked under the body and is assigned to category 3.

We photographed animals opportunistically when they were resting in a well stretched out position. We categorized all photographs according to the position of the animal using three different categories (1: well stretched out, 2: slightly curved, 3: head or pelvic region bent, see Figure 2). Animals lying completely curved were excluded from our analysis (following [21]).

To verify the accuracy of this method we compared the photogrammetric data of category 1-photographs, taken within a period of two months before or after capture, with body lengths measured during captures. Variation in body length within the same season is minimal as adult males have been shown to grow less than 5 cm per year ([29]), thus allowing a comparison of the data for the development of correction factors. We counted the number of pixels matching sea lion body length from nose to tip of tail (Image J, [30]). Using the number of pixels, body lengths were calculated based on equation (1). If several photographs were taken within 10 min of each other, the arithmetic mean was calculated to ensure independence of data. Photographs of category 1 represent the closest approximation to correct body length. For category 2 and category 3 we assessed the relative discrepancy (in %) of estimated body length to the correct body length measured during captures. To assess the repeatability of body length estimates from different photographs we calculated the coefficient of variation (CV) for body length based on individuals photographed at least three times in the reproductive season 2012. We tested the impact a deviation from the perpendicular angle has on the accuracy of our length estimates by photographing an object of 140 cm length at different angles (0.7°–13° deviation from the perpendicular angle).

We multiplied body length by girth of captured individuals and related these values to body mass (following [14]). We used linear regression to assess the fit of our proxy to adult sea lion body mass. We tested whether body height of adult sea lions estimated from photogrammetric data can be used to estimate axillary girth by the following equation (according to a circumference equation):
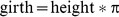
(2)


We calculated height based on equation 1 by using the number of pixels matching the body height behind the front flippers. Finally, we tested whether photogrammetric estimates of body length and height can be used to assess body mass. Again, the CV for body mass was assessed for each individual photographed at least three times in 2012. To further validate this method, we compared the values obtained from height estimates to mass estimates based on the side areas of individuals, a method which has previously been proven to provide reliable results ([21], [25]). We calculated the number of pixels equaling the side area of the animal using MATLAB (MATLAB R2011b). The contours of the animals were roughly outlined with a marker and exactly traced with an edge based segmentation technique ([31]). The side area was calculated based on a modification of equation 1 (side area [cm^2^] = (0.01395 * distance –0.00096)^2^ * number of pixels_area_). To estimate individual body mass, the side area of previously captured individuals was correlated with the measured body mass (body mass [kg] = (0.0327 * side area) –23.553, R^2^ = 0.92, [32]).

All statistical analyses were performed using R 2.15.3 ([33]). In all analyses, samples sizes refer to number of individuals, rather than the number of photos taken. We report means ± SD and the coefficient of variation (CV). Correlations are reported as Pearson’s correlation coefficients.

### Ethics statement

The research presented here, including the animal handling, complies with the animal care regulations and applicable laws of Ecuador. The work was licensed by the Galapagos National Park Service, Ecuador (Permit No PC 001-03 Ext 06-08, PC-11-08, PC 043-09, PC 0007-2011 and PC 0026-2012).

## Results

### Body length

The comparison between measurements taken at captures and body length estimates from category 1-photographs (uncorrected for the systematic errors in distance measurement) produced the following equations:
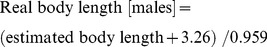
(3a)

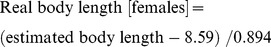
(3b)


**Figure 3:**
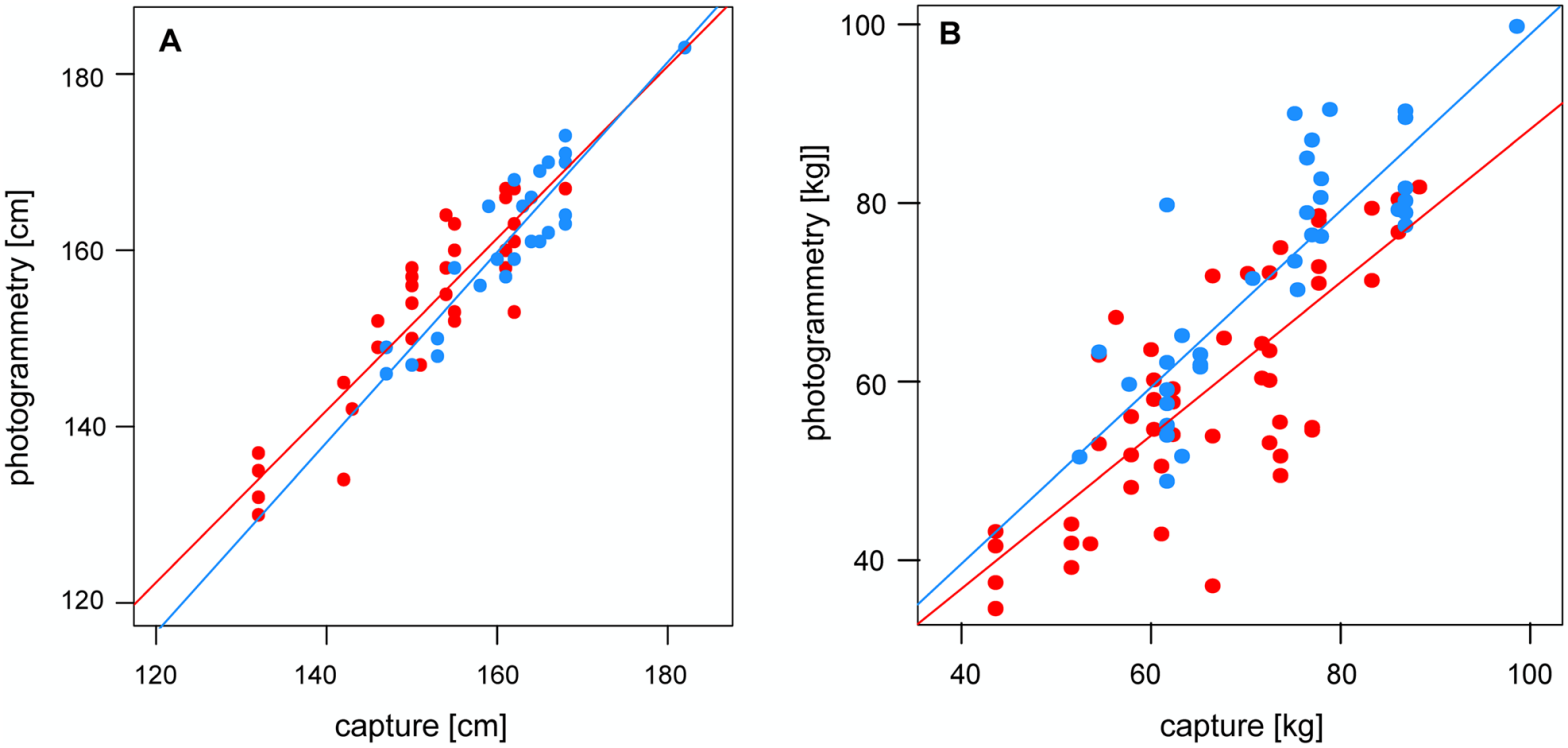
Correlation between measured and photogrammetrically estimated morphological traits (separated for males and females). Presented are the correlations between measured and estimated body length (A, category 1 - photographs) and body mass (B). Correlation coefficients differed slightly between males (blue) and females (red). Estimates varied more for mass than for length, especially in females.

**Figure 4:**
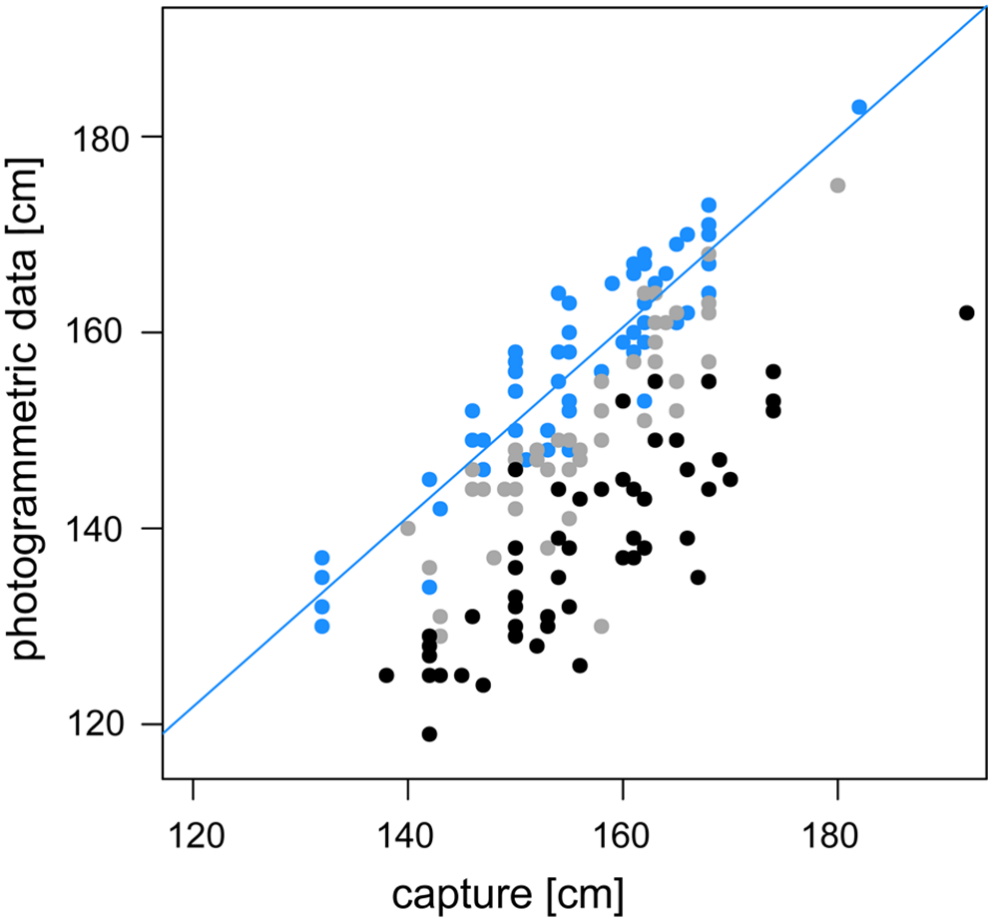
Correlation between measured and estimated body length. Measurements taken during captures and photogrammetric estimates of body length (controlled for distance error) were highly correlated. The regression line represents the relationship between both measures for photographs of category 1 (blue). Photogrammetric estimates from category 2 (grey) and category 3 (black) deviate systematically from measured body length.

**Figure 5:**
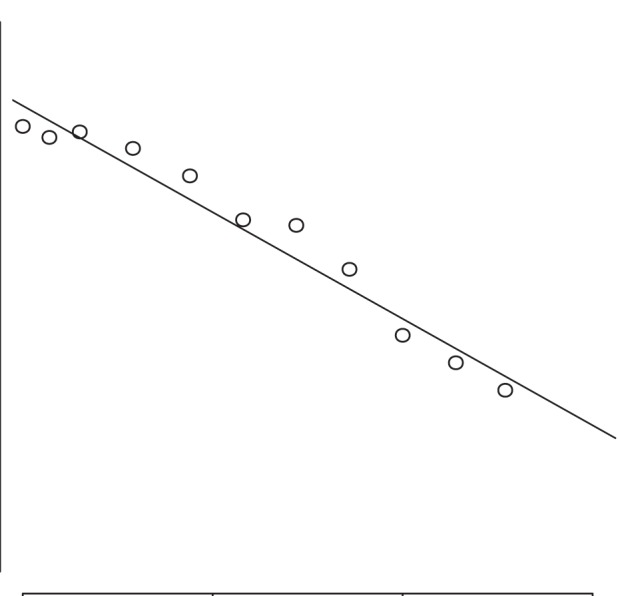
Deviation from perpendicular angle to the longitudinal axis of the animal. The graphic shows how measured length differs from real length (here 140 cm) with increasing deviation from a perpendicular angle. A deviation of 10° from the perpendicular angle leads to a reduction of 3% in length estimates.

We used these equations to calculate the correct body length estimates. We found a highly significant correlation between measured body length and estimated body length (category 1, males: N = 16, *r* = 0.921, *p*<0.001; females: N = 18, *r* = 0.898, *p*<0.001, Figure 3). The mean difference between measured and estimated body length amounted to only 3.2±1.4 cm for males (±2% of the overall mean male body length) and 4.1±2.8 cm for females (±2.6% of the overall mean female body length). The correlation between measured and estimated body length was high for each category of photos, but estimated body length differed substantially between photograph categories (Figure 4). Body length estimated from photographs of category 2 were on average 4.6±3.7% shorter than body length obtained during captures (N = 38). Estimates derived from category 3 (N = 36) were 11.8±3.4% shorter. The intra-individual CV of length estimates calculated for individuals that had at least three photographs of category 1 during the reproductive season of 2012 was 2.9% (N = 21, range: 121.2–209.2 cm). We repeated the analysis including photographs of category 2 which produced a CV of 3.7% (N = 69; adding category 3: CV = 4.8%, N = 112). Part of the variation might be explained by slight deviation in the perpendicular angle. A deviation of 10° from an angle perpendicular to an objects’ longitudinal axis leads to an underestimate of 3% in length (Figure 5).

### Body mass

To assess the relationship between the product of directly measured body length and girth and an animal’s body mass we used data for all adult individuals captured since 2003 for which standard body length, axillary girth and body mass were available (N = 175). The correlation was highly significant for males (N = 65, *r* = 0.970, *p*<0.001) and females (N = 110, *r* = 0.920, *p*<0.001). Accordingly, estimates of body mass (in kg) could be derived using morphometric measurements (in cm) when weighing proved difficult, using the following equations:
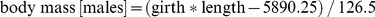
(4a)

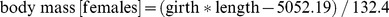
(4b)


The adjusted *R^2^* of the linear model calculated was 0.94 for males and 0.85 for females. The mean difference between measured and estimated body mass was 5.1±3.8 kg for males (±4.7% of the overall mean male body mass) and 3.9±3.2 kg for females (±6.4% of the overall mean female body mass).

To establish a photogrammetric method that allows an estimation of body mass with minimal disturbance, we tested the reliability of photogrammetric girth estimates. The correlation between measured and estimated girth was weak for animals lying in a partly curved position (category 3, LME: N = 58, *t* = −1.984, *p* = 0.050). Therefore, we excluded category 3-photographs from further analysis. In several photographs, height had to be estimated (e.g., when an animal was lying in vegetation or another individual was lying in front of it). Girth estimates derived from these photographs also tended to differ unacceptably from the capture data (LME: N = 50, *t* = −1.732, *p* = 0.086). A correction factor similar to the one introduced for body length estimates for different photograph categories, could not be implemented as the girth estimates derived from height estimates varied inconsistently. Accordingly, we excluded photographs in which height could not be estimated reliably and used only photographs that allowed unequivocal assessment of height. These data produced a highly significant correlation between girth measured and estimated from photogrammetric data (equation 2, males: N = 15, *r* = 0.787, *p*<0.001, females: N = 25, *r* = 0.718, *p*<0.001). The mean deviation from girth measurements taken during captures corresponded to 3.1±2.7 cm for males (±3.4% of mean girth) and 4.9±3.7 cm for females (±5.5% of mean girth). The intra-individual CV of girth-estimates calculated for 43 individuals for which at least three suitable photographs were available in the preproductive season of 2012 was 3.8% (range: 67.5–127.8 cm).

To estimate body mass, we only considered photographs of category 1 and 2 which enabled accurate body length estimates. We added 4.6% to the estimates derived from category 2 to estimate body length (see above). Subsequently, we multiplied body length with the corresponding girth estimate. Mass estimates were calculated using equations 4a and 4b for males and females, respectively. Measured and estimated body mass determined in this way correlated significantly (males: N = 15, *r* = 0.857, *p*<0.001; females: N = 21, *r* = 0.769, *p*<0.001, Figure 3). The mean differences between estimated and measured body mass was 5.6±4.6 kg for males and 8.8±6.7 kg for female sea lions. For males, this difference corresponded to the variation observed when calculating body mass from morphometric data measured during captures. For females, the deviation in body mass was higher when calculated from photogrammetric than from measured morphometric data. Similar results were obtained for body mass estimates calculated on the basis of the side area (males: 5.2±4.0 kg, females: 7.0±4.6 kg) demonstrating that mass estimates based on girth and length estimates perform nearly equally well. The overall intra-individual CV for body mass, calculated for 42 males and females for which suitable photographs were available in 2012, was 8.1% (range = 32.4–152.6 kg).

## Discussion

Our comparison between measurements taken during captures and photogrammetric estimates revealed a high correlation between measured and estimated morphological traits. Single-camera photogrammetry in combination with distance laser measurement provides a useful tool to obtain accurate estimates for body length and reliable estimates for body mass. Taking photographs of Galapagos sea lions even from small distances caused no visible reaction in the animals (personal observations) and captures were limited to a small number of individuals to permit calibrations. Thus, photogrammetric data collection can dramatically reduce disturbance. The CV calculated for a subset of individuals which have been photographed repeatedly within one season suggests that measurement errors were low. In addition to its accuracy, this photogrammetric method is easy to implement and has no need for expensive and bulky equipment. Given that camera and lenses are adequately calibrated, the method enables a single researcher to collect data on a large number of individuals while minimizing the stress of the animals. We stress the need for adequate calibration, because depending on the lens and camera used (focal length, lens distortion, sensor characteristics) the photogrammetry system always needs to be calibrated individually (see also Neale et al. 2011 (http://www.kineticorp.com/publications/2011-01-0286-photogrammetry-error-from-lens-distortion.pdf).

### Accuracy of body measurements

Repeated measurements of juvenile Galapagos sea lions taken during captures revealed a variance in measured individual body length of 1.3% (N = 51, unpublished data). Without anaesthesia, adult individuals are more difficult to restrain than juvenile individuals (due to their strength and larger body size). Consequently, we expect the variation in directly measured body length to be higher in adult individuals. For adult individuals, differences between measured body length and the length estimated from photogrammetry accounted for only 2% difference in males and 2.6% in females. Thus, measurement errors during captures and photogrammetric observations in our study are expected to be similar. This shows that without anaesthesia measurements obtained from captures are equally good as estimates derived from photogrammetric analysis. Further, analyses of individuals photographed multiple times within one season revealed a low CV of 2.9%. This value falls within the range of CVs found in previous studies with a minimum of 0.36% ([15]) and maximum values of 3.6% ([17]) and 3.7% ([22]), respectively. These results suggest that even small differences in body length (in the range of 5%) can be reliably estimated with our method.

Deviations between measured and estimated body mass were higher (6.8% for males and 14.5% for females). The lower accuracy obtained for body mass compared to body length can be explained by the additive effect of measurement errors while estimating body length and girth. Still, at least for males this is within the range of previous studies (3.8% in southern elephant seals: [24]; 12% in northern elephant seals: [25]). The differences found between the sexes are likely related to changes in state and body composition. Females near parturition differ in body form from non-pregnant females, with the former having the highest point not behind the fore flippers, but around the belly. This introduces additional variance into mass estimates for females. Body composition of males (and to a lesser degree of females) changes across the annual cycle due to storage of fat reserves for the reproductive season ([25], [34], [35]).

As a consequence of these potential errors, we suggest that body mass estimates should be used conservatively for categorization of individual into body mass classes rather than fine scale measurements. Nevertheless, for most researchers, obtaining mass estimates with the method presented in this study has several advantages. Only one researcher needs to approach the animal because no measuring pole needs to be placed next to the animal before taking the photograph ([14], [21]). There is no need for a second photograph to assess girth perimeter ([21], [25]) or multiple photographs to assess body volume ([23], [24]) because all required measurements can be obtained by taking one quick photograph. The incorporation of a calibration curve enables an assessment of the number of pixels that correspond to, for example, the animals’ body length, given that the distance between camera and subject is known. This decreases the time and effort needed when analyzing photographs because it overcomes the necessity to assess the number of pixels corresponding to a known distance between two fixed points separately for each photograph (two-laser photogrammetry, [22]). Thus, our method represents a time- and budget-effective technique which allows a single researcher to collect a large amount of data from many different individuals. Even if this method is insufficient to calculate energetic requirements as already mentioned by Bell and colleagues ([14]), comparison between individuals, between seasons and years are possible. This method enables scientists to investigate a variety of different research questions with regard to individual changes across the life cycle, such as health status or large changes in condition related to variance in marine productivity.

### Limitations

Notwithstanding the many advantages in getting accurate estimates for morphological traits, there are limitations to our method. However, quantifying the effect of confounding factors greatly increases the accuracy of the estimates.

We recommend calculating correction factors separately for the sexes and age classes (adults and immatures). Depending on the species, each category may differ in morphological traits and body proportions. Combining categories could lead to bias, especially for animals at the lower or upper end of the size and mass spectrum. Moreover, in -laser photogrammetry, morphological traits must be corrected for missing distances in laser distance measurements. This error originates from the laser-distance-meter measurements to the outer surface of the animal and not to the animal’s midline. This source of error must be taken into account when calculating body lengths, because ignoring this would lead to a systematic underestimation.

Additionally, not all photographs are suitable to estimate body length and mass. The position of the camera relative to the animal strongly impacts length, height, and area estimates from the photographs. In the present study, a deviation of 10° from an angle perpendicular to an objects’ longitudinal axis leads to a reduction of 3% in length. This is in accordance with previous findings of Jaquet ([16]) who found a decrease of 2.3% in length estimates. To minimize this error, researchers must make sure that photographs are taken at a 90° angle to the longitudinal axis of the subject. A potential error in the angle between camera and the animal can be avoided when using a theodolite/tachymeter instead of a simple laser distance meter, because it reports the angle and distance simultaneously.

Just like Waite et al. ([23]) we found that differences in the position of the animal caused deviations in body length. Pinnipeds, especially otariids, are highly flexible in their movements, and even when lying on a flat surface, these animals are often found in curved position. Length estimates from photographs of individuals lying in a curved position resulted in underestimates of body length. We categorized photographs depending on the degree of body curvature and calculated the difference between actual body lengths and body length obtained for the different photograph categories. Adding the discrepancy to the original estimates provided an unbiased adjustment of measurements from photographs with a curved position of an animal.

Depending on the purpose of each study the inclusion of photographs where individual position is curved or deviates from perpendicular angle to the camera may be considered, but in this case, a lower accuracy of the estimated morphological trait has to be tolerated. Thus, the single-photogrammetric method can be extended to animals that are able to flex, stretch out, and contract their body. To obtain highly accurate estimates for body length and body mass, photographs should be chosen taking these issues into account. Furthermore, when applying this method to different species, one must consider that distance measurements are limited by the ability to correctly point the laser on the subject, either because the object is too small or distances are too large. Because the laser and lens are firmly attached next to each other, this should only become a problem with very small species (i.e., smaller than the distance between laser and lens) or at very long distances. Breuer and colleagues ([15]) have shown that depending on the visibility within the habitat, accurate distance measurements are possible for more than 400 m. However, measurement errors must be less than inter-individual differences within the species to obtain useful results.

Researchers have to keep in mind that depending on the characteristics of camera and lenses used for photogrammetric studies, pixel sizes at the center may differ from the ones at the edge of an image leading to orthogonal distortion. This may cause a problem with the reliability of the estimates. In the present study, we used a 55 mm lens to minimize distortion, were able to photograph animals in such a way that they largely filled the frame and carefully calibrated the system by photographing a standard object at different distances. Further, adult Galapagos sea lions show little variation in body size (120–210 cm). Accordingly, the calibration we conducted with a 1 m pole proved sufficient to assess body length accurately. However, orthogonal distortion will become an issue if animals cannot be approached so easily and lenses of different focal length must be used or when a species varies much more in body length.

### Conclusions

Given the above mentioned caveats, our method, if properly validated for different lenses, cameras and species, provides an excellent opportunity to collect morphological data for large numbers of individuals with minimal disturbance and low financial cost. This is a great advantage considering that the stress caused by captures can lead to undesirable consequences. Additionally, on an individual level, it allows researchers to determine changes in body condition across the year, with growth or with changes in food abundance.
